# Characterization of a Toothpaste Containing Bioactive Hydroxyapatites and In Vitro Evaluation of Its Efficacy to Remineralize Enamel and to Occlude Dentinal Tubules

**DOI:** 10.3390/ma13132928

**Published:** 2020-06-30

**Authors:** Lorenzo Degli Esposti, Andrei C. Ionescu, Eugenio Brambilla, Anna Tampieri, Michele Iafisco

**Affiliations:** 1 Institute of Science and Technology for Ceramics (ISTEC), National Research Council (CNR), Via Granarolo 64, 48018 Faenza, Italy; lorenzo.degliesposti@istec.cnr.it (L.D.E.); anna.tampieri@istec.cnr.it (A.T.); 2 Oral Microbiology and Biomaterials Laboratory, Department of Biomedical, Surgical, and Dental Sciences, University of Milan, Via Pascal, 36, 20133 Milan, Italy; andreiionescu_40@hotmail.com (A.C.I.); eugenio.brambilla@unimi.it (E.B.)

**Keywords:** toothpaste, remineralization, enamel, dentin, caries, hydroxyapatite, fluoride, dental hypersensitivity

## Abstract

Demineralization of dental hard tissues is a well-known health issue and the primary mechanism responsible for caries and dentinal hypersensitivity. Remineralizing toothpastes are nowadays available to improve conventional oral care formulations regarding the prevention and repair of demineralization. In this paper, we analyzed the chemical-physical features of a commercial toothpaste (Biosmalto Caries Abrasion and Erosion, Curasept S.p.A., Saronno, Italy), with particular attention paid to the water-insoluble fraction which contains the remineralizing bioactive ingredients. Moreover, the efficacy of the toothpaste to induce enamel remineralization and to occlude dentinal tubules has been qualitatively and semiquantitatively tested in vitro on human dental tissues using scanning electron microscopy and X-ray microanalysis. Our results demonstrated that the water-insoluble fraction contained silica as well as chitosan and poorly crystalline biomimetic hydroxyapatite doped with carbonate, magnesium, strontium, and fluoride ions. The formulation showed excellent ability to restore demineralized enamel into its native structure by epitaxial deposition of a new crystalline phase in continuity with the native one. It was also able to occlude the dentinal tubules exposed completely by acid-etching. Overall, this study demonstrated that the tested toothpaste contained a biomimetic ionic-substituted hydroxyapatite-based active principle and that, within the in vitro conditions analyzed in this study, it was effective in dental hard tissue remineralization.

## 1. Introduction

Demineralization of dental tissues is an issue affecting an increasingly large percentage of the population worldwide, and the main mechanism involved in the development of dental caries and dentin hypersensitivity [1]. Demineralization is caused by low pH values reached by the oral microenvironment. It is the outcome of very complex interactions that take place between hard tissues, the microbial biofilm that consistently colonizes these surfaces and the intake of nutrients, other situations such as the consumption of acidic food or drinks (i.e., fruits, coffee, tea, soft drinks, sports drinks, and fruit juices), and the presence of gastroesophageal reflux disease [2]. When the pH drops below ≈5.5, the process of dissolution of hydroxyapatite (HA), which is the mineral part representing 95 wt% and 75 wt% of enamel and dentin, respectively, takes place [3,4]. It has to be noted that a cariogenic biofilm can reach pH values lower than 4.0 close to the surface [5]. In its early steps, demineralization is a reversible process if the damaged tissues are exposed to an oral environment that favors remineralization [6]. For example, the calcium and phosphate ions naturally present in the saliva counteract demineralization, as they favor epitaxial growth starting from residual HA prisms [6]. However, when hard tissues face a significant increase in the presence of acids, the remineralizing action of saliva is not sufficient, and a progressive loss and weakening of the mineral phase take place, ultimately leading to carious lesions [1]. It has been demonstrated that small reductions in lowest pH values reached by biofilms or selective inhibition of the metabolism of the aciduric populations, such as the ones obtained by the release of fluoride and other ions, could avoid pathogenic bacteria to outcompete beneficial ones [7,8]. In this sense, small but accumulative effects can prevent dysbiotic changes in dental biofilms and can help maintain a healthy oral microbiome. Therefore, it could be beneficial to both the host and its biofilm to use proper external agents to trigger the remineralization and to prevent demineralization processes. Toothpastes are considered the most effective and affordable vehicles of remineralizing agents, and the incorporation of bioactive agents to contrast enamel and dentin erosion in toothpastes has become increasingly common [9,10].

Fluoride is considered the gold standard in the inhibition of the demineralization processes [9]. Its well-known effects are directed both on the reduction of metabolic and physiological pathways of acidogenic microorganisms and on partially replacing the hydroxyl groups in HA crystals, inducing the formation of fluorapatite. The latter is less soluble and thus more resistant to low pH values than pristine HA [11,12]. The administration of fluoride-containing treatments has to be, however, carefully balanced in order to avoid possible side effects such as dental fluorosis or a negative impact on tooth development [13].

Chitosan, a natural polymer obtained by deacetylation of chitin, is an interesting compound used in the prevention of dental demineralization as it provides bactericidal or bacteriostatic characteristics [14,15]. Under acidic conditions, the amino groups of chitosan capture hydrogen ions, resulting in an overall positive charge that confers to the molecule adhesive ability by electrostatic forces to negatively charged surfaces such as tooth enamel [16]. Therefore, chitosan acts as a tamper layer, modulating the penetration of acidic compounds as well as inhibiting the release of ions from the hard tissues [14,16]. Furthermore, since several bacterial species produce enzymes that can dismantle chitosan over time depending on its molecular weight and degree of deacetylation [17], it can be envisaged as a promising tool to obtain targeted delivery of ions and active principles to tooth surfaces for a defined amount of time [18].

Nowadays, synthetic HA is commonly accepted as a remineralizing agent, and its effectiveness is widely described and validated in vitro and in vivo in the scientific literature [19,20,21,22,23]. Recently, Enax et al. revised the modes of action of HA in preventive oral health care [3]. They identified different activities of HA in the oral cavity: (i) physical restoration, that is the attachment of HA particles to the tooth surface and cleaning properties; (ii) biochemical effect by releasing calcium and phosphate ions under acidic conditions forming an interface between HA particles and the enamel; and (iii) biological interaction of HA particles with microorganisms [3]. In addition, HA-based materials can desensitize exposed dentinal tubules by occluding them, thus forming a mineralized barrier [24,25].

This work aimed to perform a chemical-physical characterization of a novel commercially available toothpaste (Biosmalto Caries Abrasion and Erosion of Curasept S.p.A, Saronno, Italy), with particular attention paid to the water-insoluble fraction which, according to the manufacturer, contains all the remineralizing bioactive ingredients described above, such as ion-doped hydroxyapatite (Sr-Mg-CO_3_-HA) and HA partially substituted with fluoride ions (FHA) embedded in a chitosan matrix. Also, we have qualitatively and semiquantitatively evaluated the ability of this toothpaste to remineralize enamel and to occlude exposed dentine tubules in vitro. This study is the first public report documenting the characteristics and the functionalities of this particular toothpaste.

## 2. Materials and Methods

### 2.1. Materials

The test toothpaste was Biosmalto Caries Abrasion and Erosion (lot number 101/9, expiry date 02/2022), having a nominal fluoride content of 1450 ppm, and it was provided by Curasept S.p.A., Saronno (VA), Italy. The ingredients are shown in Table 1. Modified Dulbecco’s phosphate-buffered saline (Dulbecco’s PBS, without CaCl_2_ and MgCl_2_), orthophosphoric acid (H_3_PO_4_ 85 wt% in H_2_O), hydrochloric acid (HCl 37 wt% in H_2_O), and hydrofluoric acid (HF 40 wt% in H_2_O) were purchased from Sigma Aldrich (St. Luis, MO, USA). All the solutions were prepared with ultrapure water (18.2 MΩ × cm, 25 °C, Arium© pro, Sartorius, Göttingen, Germany).

### 2.2. Sample Preparation

In order to collect and analyze the water-insoluble fraction, the toothpaste was washed with ultrapure water. The washing procedure consisted in the dispersion of 5 g of the toothpaste in 150 mL of ultrapure water, followed by repeated washings with ultrapure water by centrifugation at 10,000 rpm for 5 min. Finally, the washed product was freeze-dried overnight at −50 °C under a vacuum of 3 mbar, and the lyophilized powders were subsequently ground and sieved with a 50 μm sieve in order to achieve a uniform granulometry. The dried powder was weighted to quantify the water-insoluble fraction of the toothpaste. An aliquot of this powder was also dissolved with a 6 wt% HNO_3_ solution in order to collect the acid-insoluble fraction that represented the total silica content. The latter fraction was collected by centrifugation, freeze-dried overnight, and then weighted. Also, the whole toothpaste was freeze-dried overnight and then weighted in order to estimate its water content.

### 2.3. Structural Characterization

Powder X-ray diffraction (PXRD) patterns of the powdered toothpaste samples were recorded using a D8 Advance diffractometer (Bruker, Karlsruhe, Germany) equipped with a Lynx-eye position-sensitive detector using Cu Kα radiation (λ = 1.54178 Å) generated at 40 kV and 40 mA. PXRD patterns were recorded in the 2 θ range from 10–60° with a step size (2θ) of 0.02° and a counting time of 0.5 s.

Fourier transform infrared (FTIR) spectroscopy analyses of the sample were carried out on a Nicolet iS5 spectrometer provided with attenuated total reflectance (ATR) accessory iD7 (Thermo Fisher Scientific Inc., Waltham, MA, USA) with a resolution of 4 cm^−1^ by an accumulation of 32 scans in ATR mode.

### 2.4. Compositional Analysis

Quantification of Ca, P, Mg, Sr, and Si was carried out by inductively coupled plasma atomic emission spectrometer (ICP-OES) (Agilent Technologies 5100 ICP-OES, Santa Clara, CA, USA). Both the water-insoluble fraction and the whole toothpaste were analyzed. Samples from water-insoluble fraction were prepared by dissolving 100 mg of powder in 50 mL of a 0.8 wt% HF solution.

Fluorine presence in the whole toothpaste and the water-soluble fraction was quantified with a fluoride ion electrode (Intellical™ ISEF121, Hach Lange, Loveland, CO, USA). In the first case, an aliquot of the toothpaste (1 g) was dispersed in 100 mL of ultrapure water, and then, a 6 mL aliquot of this suspension was mixed with an equal volume of 37 wt% HCl for 1 h at 50 °C in order to completely dissociate fluoride from monofluorophosphate ions and FHA [26]. Afterwards, the acid-insoluble components were separated by centrifugation (14,000 rpm for 3 min), and the supernatant was analyzed using the protocol suggested by the instrument’s manufacturer. To quantify the fluorine content in the water-soluble fraction of the toothpaste, an aliquot of the toothpaste (1 g) was dispersed in 100 mL of ultrapure water and the water-insoluble component was separated by centrifugation (14,000 rpm for 3 min). The supernatant was treated with 37 wt% HCl and then analyzed according to the protocol suggested by the instrument’s manufacturer.

Thermogravimetry analysis (TGA) of the freeze-dried toothpaste and the water-insoluble fraction was recorded using an STA 449F3 Jupiter (Netzsch GmbH, Selb, Germany) apparatus. About 10 mg of sample was weighted in an alumina crucible and heated from room temperature to 1100 °C under airflow with a heating rate of 10 °C/min.

### 2.5. In Vitro Evaluation of Remineralization and Dentinal Tubules Occlusion

In vitro experiments were performed on sound human molar teeth extracted for clinical reasons (Oral Surgery Unit, Department of Biomedical, Surgical and Dental Sciences, Milan, Italy). The Institutional Review Board of the University of Milan approved the use of the teeth (protocol SALTiBO–2017), and written informed consent was obtained from each donor. This part of the study was performed according to the principles of the Declaration of Helsinki updated by the World Medical Association in 2013.

**Figure 1:**
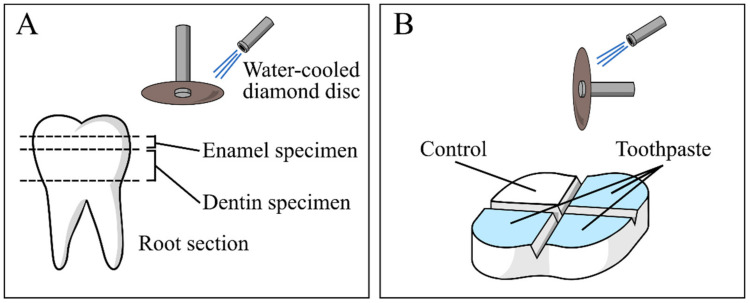
Schematic representation of specimen preparation: (**A**) horizontal sections of tooth in order to expose enamel and dentin and (**B**) delimitation of control and treatment regions.

A total of three teeth specimens were sectioned horizontally under constant water cooling at two levels in order to cut away the root 2 mm apical to the cementoenamel junction, and to expose flat enamel (n = 12) or dentin (n = 12) surfaces. A low-speed diamond disc (Horico, Berlin, Germany) mounted on a universal drill and drill hold (Robert Bosch GmbH, Gerlangen, Germany) was used (Figure 1A). Then, four regions were delimited on the top surface of each specimen (either dentine or enamel) by making two perpendicular 0.5 mm deep notches using the same disc (Figure 1B). After that, specimen surfaces were polished using silicon carbide paper (600 and 1200 grit) and the surfaces were demineralized using a 37 wt% H_3_PO_4_ gel (Gluma Gel, Heraeus Kulzer, Hanau, Germany), which was applied for 30 s, followed by extensive rinsing with ultrapure water [27].

In vitro enamel remineralization and dentinal tubule occlusion experiments were performed on eighteen demineralized specimens (nine of enamel and nine of dentin) that were treated with the toothpaste by soft brushing for 3 min, followed by extensive rinsing with ultrapure water. Treatment was carried out three times per day and repeated for three days, keeping the specimens in fresh Dulbecco’s PBS at 37 °C between treatments. This storage solution was selected because it does not contain calcium ions, in order to avoid a possible influence of these ions on the remineralization process. After the third day, the specimens were rinsed with ultrapure water and dried at 37 °C. A total of six demineralized specimens (three of enamel and three of dentin) that were only brushed with ultrapure water were used as negative controls.

Afterward, all specimens were mounted on stubs with conductive tape, sputter-coated with gold (Polaron Sputter Coater E5100, Polaron Equipment, Watford, Hertfordshire, UK) and observed with a field-emission scanning electron microscope (FEG-SEM, mod. ΣIGMA, ZEISS NTS Gmbh, Oberkochen, Germany) at 10 kV acceleration voltage. For each region of a given specimen, four randomly selected fields were recorded at magnifications of 5000×, 25,000× and 50,000× (enamel) or 2500× and 10,000× (dentin).

Energy-dispersive X-ray spectroscopy (EDX) analysis was performed using a TM4000Plus Tabletop scanning electron microscope (Hitachi, Schaumburg, IL, USA) equipped with an EDX probe (Q75, Bruker, Berlin, Germany) to investigate the elemental composition of the specimen surface. Dry specimens were observed in surface-charge reduction mode without sputter-coating, using an accelerating voltage of 15 kV. Three randomly selected fields were acquired for each region of a given specimen at 300× magnification and were analyzed using the EDX probe in full-frame mode using an acquisition time of 150 s. Two additional tooth specimens were sectioned at enamel level, then polished, and demineralized, and one half of the surface was treated with the tested toothpaste as previously specified while the other half was left demineralized (control surface). When brushing, extra care was taken not to touch the control surface with the tested toothpaste, and rinsing was performed on inclined specimens with the control surface oriented upwards. These specimens were dried and observed using EDX in map mode. Three fields were acquired, observing the interface between treated and non-treated surface at 300× and 1000× magnification with an acquisition time of 10 min to highlight topographical differences in the elemental composition of the surface. All acquired data represent the elemental composition of the ≈1 μm superficial layer.

### 2.6. Statistical Analysis

All experiments regarding the structural characterization and compositional analysis were performed in triplicate and repeated at least three times. Data are reported throughout the text as means ± 1 standard error.

## 3. Results and Discussion

### 3.1. Structural Characterization

**Figure 2:**
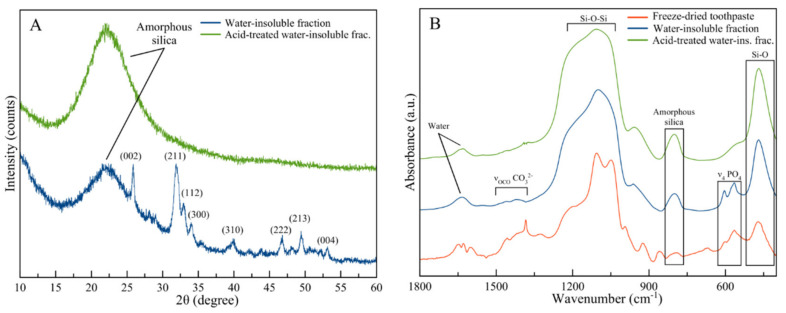
(**A**) Powder X-ray diffraction (PXRD) patterns and (**B**) Fourier transform infrared (FTIR)–attenuated total reflectance (ATR) spectra of the total toothpaste, the water-insoluble fraction, and the acid-treated water-insoluble fraction.

The washing process separated the water-insoluble phase, which, according to our results, accounts for 15 ± 1 wt% of the toothpaste. The remaining part was composed of water (52 ± 7 wt%) and water-soluble organic substances (33 ± 3 wt%). The PXRD pattern of the water-insoluble fraction of the toothpaste shows the typical diffraction peaks of HA as the main crystalline phase (PDF card file 00-009-0432) (Figure 2A). In particular, the most intense reflections are the narrow peak at 25.87° due to the (0 0 2) crystallographic planes and the broad peak centered at about 32° that is the sum of three peaks at 31.77°, 32.19°, and 32.90° due to planes (2 1 1), (1 1 2), and (3 0 0). Other peaks are present at 39.81°, 46.71°, 49.46°, and 53.14°, which correspond to the planes (3 1 0), (2 2 2), (2 1 3), and (0 0 4), respectively [28]. The diffraction peaks of HA are broad and poorly defined, indicating a reduced degree of crystallinity of this phase, thus showing a good similarity with biogenic HA [29,30]. A broad band centered at 23° was also observed, suggesting the presence of an amorphous phase, which corresponds to silica [31]. Therefore, PXRD has revealed that the water-insoluble fraction of the toothpaste is composed of amorphous silica and poorly crystalline HA, in agreement with the list of ingredients (Table 1).

It is important to remark that the diffraction peaks reported in Figure 2A are the result of both crystalline phases contained in the toothpaste, i.e., Sr-Mg-CO_3_-HA and FHA. As already reported, these two phases produced almost identical diffraction patterns [32,33]; therefore, it was not possible to distinguish the single components and, thus, their relative abundance. The other characterization techniques, as well as the separation methods used in this work, were not able to distinguish between Sr-Mg-CO_3_-HA and FHA. Consequently, for the sake of simplicity, this mixture will be referred to as one single HA phase throughout the discussion.

PXRD was also collected on the acid-insoluble fraction of the powder. As expected, the pattern was composed only by the broad band centered at 23° that was attributed to amorphous silica, indicating that the acidic treatment has dissolved the HA phase completely.

Complementary analyses to PXRD were carried out using FTIR–ATR spectroscopy (Figure 2B). The analysis was carried out comparing the spectra acquired on the whole freeze-dried toothpaste, on the water-soluble fraction, and on this latter after acid treatment.

In the freeze-dried toothpaste, the presence of organic components can be assessed by the vibrations of their functional groups in the domain around 650–900 cm^−1^ and 1300–1600 cm^−1^. The bands related to the organic components disappeared in the spectrum of the inorganic fraction, where the most intense bands of the sample are due to Si–O bond vibration of silica. In particular, these are visible at around 455 cm^−1^ (Si–O rocking) and 1055 cm^−1^ (Si–O–Si siloxane vibration) together with weak bands at ca. 1220 and 800 cm^−1^ that are typical of amorphous silica [34]. The two bands at 603 and 565 cm^−1^ are due to the triply degenerated bending mode of PO_4_ groups (ν_4_PO_4_) of HA. Two weak bands in the 1350–1500 cm^−1^ range were attributed to stretching vibrations of carbonate group (ν_3_CO_3_) and confirm the presence of carbonate ions that can be found as doping ions of HA phase in good consistency with the ingredients list of the toothpaste (Table 1). These bands are broad and weak, making it relatively challenging to evaluate their chemical nature. However, the position of carbonate peaks (ca. 1415 cm^−1^ and ca. 1460 cm^−1^) suggests that they are a partial substitute for PO_4_^3−^ ions (carbonate substitution type B) of HA [35].

Chitosan is insoluble in water but soluble in acid medium. However, we did not detect the most intense signals related to this molecule in the spectrum of the water-insoluble fraction, which are at ca. 700–1100 cm^−1^ [36], probably because they are overlapped with those of silica and HA. The signals of PO_4_ and carbonate groups disappeared in the spectrum of the inorganic components after acid treatment, confirming that carbonate ions are included in HA phase. The water band centered at about 1640 cm^−1^ is observed in all the spectra, witnessing the still hydrated character of these “dried” samples.

### 3.2. Compositional Analysis

The chemical composition of the toothpaste is reported in Table 2. The quantification was carried out on the whole toothpaste and on the water-insoluble fraction. The single components of this latter phase (i.e., HA and silica) were quantified by weight, dissolving the HA phase in nitric acid and removing the silica, which is insoluble in acid, by centrifugation. It was estimated that the water-insoluble fraction is composed of 10 ± 1 wt% silica and 5 ± 0.5 wt% HA.

Chemical analysis of the whole toothpaste showed the presence of Si, Ca, P, and F as the most abundant elements and the presence of Mg and Sr in minor quantity. The quantities of Si and Ca are in agreement with the content of silica and HA reported above. The total fluorine content of the toothpaste due to the contribution of both FHA and mono-fluorophosphate ions corresponds to 1458 ± 25 ppm, which is in agreement with the declared value (1450 ± 50 ppm). The fluorine content of the water-insoluble fraction, i.e., FHA only, was quantified to be 1390 ± 25 ppm. This value was obtained by subtracting the quantity of fluorine contained in the water-soluble fraction, which was due to mono-fluorophosphate ions only. The high content of fluorine in the solid fraction shows that FHA is the primary fluorine source of the toothpaste and that FHA is the most abundant phase among the HA components, in agreement with the ingredients list.

Elemental analyses of the water-insoluble fraction of the toothpaste confirmed the results of PXRD and FTIR-ATR measurements, indicating Si as the main component (29.8 wt%), followed by Ca (9.2 wt%), and P (4.6 wt%). The relative abundance of these three elements further confirms the silica/HA composition ratio reported above. The presence of Mg and Sr suggests that Mg^2+^ and Sr^2+^ ions are present as doping ions of HA. The calcium to phosphate molar ratio (Ca/P) of the solid fraction is 1.51, which is lower than the stoichiometric ratio of HA (1.67), but assuming a substitution of Ca^2+^ by Mg^2+^ and Sr^2+^, the ratio (Ca + Mg + Sr)/P is 1.61, which is closer to the stoichiometric Ca/P value, corroborating the possible doping of magnesium and strontium in HA as reported in the ingredients list. Caution should be taken in interpreting these data because, as already mentioned, it was not possible to distinguish between Sr-Mg-CO_3_-HA and FHA.

**Figure 3:**
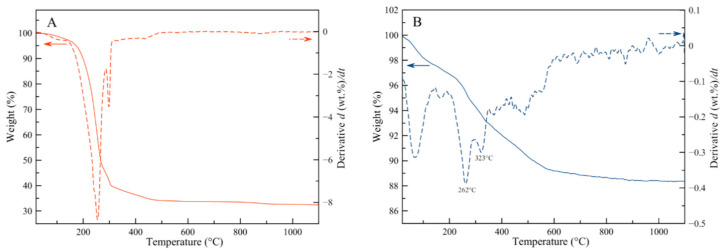
Thermogravimetry analysis (TGA)–derivative thermogravimetric (DTG) curves of (**A**) the freeze-dried toothpaste and (**B**) the water-insoluble fraction.

Thermogravimetry analyses (TGA) were carried out to have an overview of the components of the freeze-dried toothpaste and the water-insoluble fraction (Figure 3). The TGA curve of the freeze-dried toothpaste (Figure 3A) showed a sharp weight loss of about 60 wt%, occurring between 200 °C and 400 °C. This weight loss was attributed to the thermal degradation of the organic components. The organic/inorganic weight ratio estimated by TGA was 70/30 that is in agreement with the value calculated weighing the specimens after the washings. The TGA curve of the water-insoluble fraction (Figure 3B) showed a gradual weight loss of about 12 wt% from room temperature to 1100 °C. Two main variations in weight loss were identified. The first one of about 3 wt% took place between room temperature and 200 °C and was due to the removal of adsorbed water. The second one of about 8 wt% occurred between 200 °C and 400 °C. In this case, the derivative thermogravimetric (DTG) curve showed the presence of two thermal events at 262 °C and at 323 °C that were both related to the thermal degradation of chitosan macromolecule [37]. Overall, this datum indicates that the water-insoluble fraction of the toothpaste contained about 8 wt% of chitosan.

### 3.3. In Vitro Evaluation of Remineralization

HA was associated with chitosan to improve the attachment and dwelling time on dental surfaces, thanks to the adhesive properties of the biopolymer. The use of FHA instead of fluoride salts was seen as a long-term topical delivery of fluoride directly in the dental cavities/lesions, potentially enhancing its efficacy and reducing possible side effects due to excessive intake of fluoride that might lead to dental or skeletal fluorosis.

**Figure 4:**
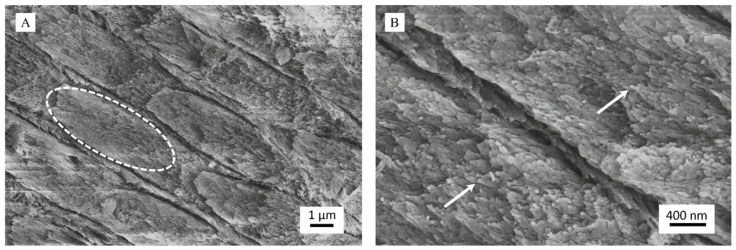
Representative field-emission scanning electron microscope (FEG-SEM) micrographs of demineralized dental enamel after control treatment (water only) at magnification levels of (**A**) 25,000× and (**B**) 50,000×: In (**A**), an enamel prism is enclosed in a white ellipse. In (**B**), single native hydroxyapatite (HA) nanocrystals are marked by white arrows.

The efficacy of the toothpaste as an enamel remineralizing product was tested on human dental enamel specimens that were demineralized with 37 wt% H_3_PO_4_ following a well-established protocol. After acid etching, control samples (Figure 4) appeared utterly eroded, and the individual prisms could be seen well separated due to the corrosion of the interprismatic mineral phase (Figure 4A). It was also possible to observe each native HA crystal that constitutes the primary unit of enamel prisms since etching had dissolved the outermost amorphous enamel layer (Figure 4B). Therefore, acid etching successfully demineralized enamel surfaces. As expected, the control treatment, albeit providing a source of phosphate due to the storage in Dulbecco’s PBS, did not show any remineralization effect. Dulbecco’s PBS was used as a storage medium in the present study to ensure that an external source of calcium was not provided to the teeth, thus excluding a possible confounding factor when assessing the remineralizing capacity of the toothpaste. Further studies may be performed, including a closer simulation of the oral environment, such as the use of artificial saliva and an oral cariogenic biofilm developed over the surfaces of the specimens using a bioreactor.

**Figure 5:**
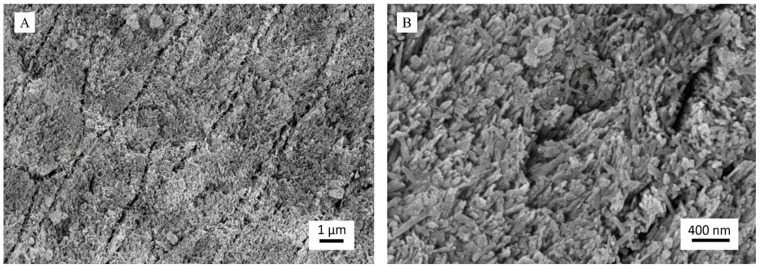
Representative FEG-SEM micrographs of demineralized dental enamel after treatment with the toothpaste at magnification levels of (**A**) 25,000× and (**B**) 50,000×.

The demineralized specimens treated with the toothpaste presented a very different surface morphology (Figure 5). Specifically, it was still possible to observe the prismatic enamel structure, but the prisms were not separated, as it was more challenging to distinguish their limits. This feature indicates that the toothpaste has remineralized the interprismatic spaces by depositing a new mineral phase (Figure 5A). At higher magnification, FEG-SEM images clearly revealed the presence of new crystals, about 100 nm long, having the same orientation as the pristine crystals (epitaxial growth) and homogeneously growing on prisms and in the interprismatic spaces (Figure 5B). It is important to stress that the new crystals were less ordered than the native ones. However, they were not randomly distributed on the eroded enamel but orientated following the directions of the natural HA crystals.

Through synoptic comparison of control and treated specimens, it is evident that the repeated application of the toothpaste on a short timescale has led to the remineralization of the eroded enamel by depositing a new crystalline phase in continuity with the native one.

Semiquantitative EDX compositional analysis was carried out on control and toothpaste-treated specimens in order to study the elemental composition of the enamel surface layer (≈1 μm, Table 3, and Figure S1). The EDX analysis on vacuum-dried whole toothpaste was also performed, evincing a chemical composition in agreement with the above-reported data (Figure S1A and Table S1). The enamel control specimens showed the presence of C, Ca, P, Na, Mg, and Cl (Figure S1B). Traces of Al and Si come from the polishing procedures despite thorough cleaning and acid treatment of the surface. The Ca/P molar ratio was 1.79 ± 0.02, which is typical of enamel hydroxyapatite [38,39]. Specimens treated with the toothpaste showed the presence of the same elements and have a similar Ca/P ratio (1.89 ± 0.01); however, fluorine and strontium were also detected (ca. 0.2–0.4 wt% and 0.2–0.3 wt%, respectively, Figure S1C). The different surface morphology, an increased presence of Si, and the presence of F and Sr indicated that a new mineral phase had been deposited on eroded enamel. This new mineral phase had a similar chemical composition to biogenic apatite but enriched with fluorine and contained silica derived from the toothpaste as well. EDX elemental mapping (Figure S2) showed that the topographical distribution of elemental composition was generally very similar between enamel surfaces treated with the toothpaste and control demineralized surfaces. The presence of Si- and Sr-rich clusters with a mean diameter of 30 μm (Figure S2C) was highlighted on enamel surfaces after toothpaste treatment. However, these observations are restricted by the limits of the EDX elemental mapping technique that include a relatively low spatial resolution in mapping mode and low signals for F, Sr, Zn, and Mg due to amounts close to the detection limit of the instrument.

### 3.4. In Vitro Evaluation of Dentinal Tubules Occlusion

**Figure 6:**
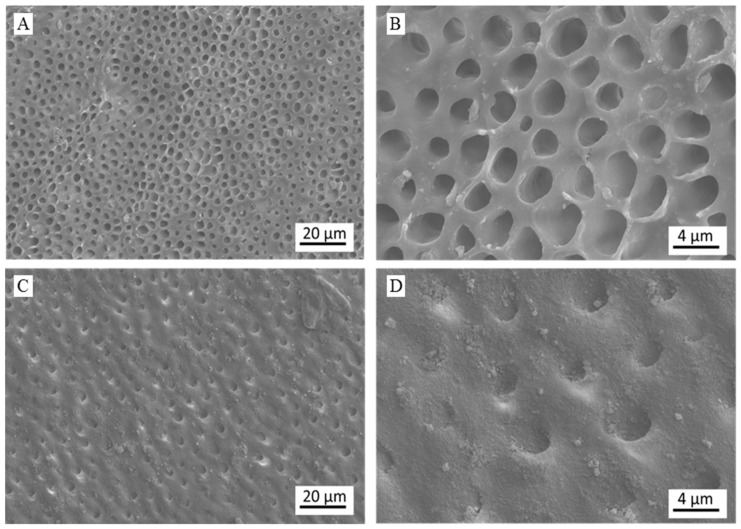
Representative FEG-SEM micrographs of demineralized dentine treated with (**A**,**B**) only water and (**C**,**D**) the toothpaste (below) at magnification levels of (**A**,**C**) 2500× and (**B**,**D**) 10,000×.

The effectiveness of the toothpaste as a dentinal tubule occluding agent was tested on human dentine specimens demineralized with concentrated phosphoric acid. After acid etching, the control surfaces showed open and enlarged dentinal tubules without any evidence of occlusion (Figure 6A,B). After treatment with the toothpaste, the surface morphology of the specimen was utterly changed (Figure 6C,D). Specifically, a new crystalline phase was deposited, which caused the complete occlusion of exposed dentinal tubules.

Semiquantitative EDX compositional analyses were carried out on the control and on the treated specimens. For the control samples, only the presence of C, O, and N has been revealed. This finding suggests that, after the demineralization process, only the organic component of dentine (i.e., mainly collagen) was preserved while the surface mineral component was totally dissolved by acid etching. In the tested specimen, the contents of nitrogen and oxygen were 25 ± 1 wt% and 31 ± 1 wt%, respectively. EDX analysis carried out on the specimens treated with the toothpaste revealed the presence of the same elements previously detected on the control (N: 10.8 ± 1.3 wt%, O: 37 ± 1 wt%) together with Si (4.4 ± 0.8 wt%), Ca (1.0 ± 0.2 wt%), and P (0.7 ± 0.1 wt%). The Si/Ca/P ratio measured on specimens treated with the toothpaste (5.7/1.4/1) was very similar to the ratio of the inorganic phase of the toothpaste (6.3/1.9/1), suggesting an effective deposition of this solid phase on dentine surface. The decrease in the intensity of N signal and the increase in the intensity of O signal are other findings that corroborate the formation of a new mineral phase. Overall, the surface morphology and chemical composition analyses suggest that dentin was covered by a new toothpaste-derived mineral phase composed of silica and HA, where the latter remineralized the dentinal tissue and completely occluded the exposed dentinal tubules.

On the basis of the preliminary in vitro tests performed herein, the toothpaste appears to have an effective remineralizing activity. These data are encouraging and worthy of future in vivo clinical investigations aimed at confirming that the daily use of this toothpaste significantly leads to remineralization of enamel and a reduced dentinal hypersensitivity.

## 4. Conclusions

The aim of this work was to study a commercially available toothpaste that claimed to have a remineralizing effect. PXRD, FTIR-ATR, and chemical characterizations have evinced that the inorganic water-insoluble fraction of the toothpaste bearing the bioactive compounds was composed of amorphous silica and poorly crystalline biomimetic HA doped with CO_3_^2−^, F^−^, Mg^2+^, and Sr^2+^ ions. The presence of chitosan as a macromolecular component in the water-insoluble fraction was evaluated by TGA-DTG.

The efficacy of the toothpaste to induce enamel remineralization and to occlude dentinal tubules has been qualitatively and semiquantitatively tested in vitro by FEG-SEM and EDX analyses. The treatment of the demineralized specimens of enamel and dentine with the toothpaste produced a mineral deposit on the surface of the specimens that led to the occlusion of dentinal tubules and filling of enamel interprismatic spaces, restoring the depleted mineral content induced by acid etching. Therefore, the reported experiments proved that the toothpaste was effective as a remineralizing product, as claimed by the manufacturer, even after a short treatment time (three days), due to the deposition of HA (chemically similar to biogenic HA) onto depleted enamel prisms and exposed dentinal tubules. A direct correlation between diameter and density of open dentinal tubules and teeth hypersensitivity is well reported in the literature [24,40,41]. Although the data presented in this work are in vitro results, it can be speculated that the high degree of dentinal tubules occlusion exerted by the toothpaste could lead to a desensitizing clinical effect. Additional investigations have to be carried out in the future both to clinically study the effect of the toothpaste on enamel and dentin on the long-term scale and to better characterize the exact nature of the new deposited mineral phase on the tooth surface by more advanced and sensitive analytical techniques.

## Figures and Tables

**Table 1:** Ingredients list of the test toothpaste.

Commercial Name	Ingredients
Biosmalto Caries Abrasion and Erosion	Purified water, glycerin, hydrated silica, fluorohydroxyapatite, magnesium-strontium-carbonate-hydroxyapatite conjugated with chitosan, cellulose gum, xylitol, cocamidopropyl betaine, xantham gum, aroma, sodium monofluorophosphate, potassium acesulfame, ethylhexylglicerin, phenoxyethanol, sodium benzoate, citric acid.

**Table 2:** Chemical composition of the whole toothpaste and the water-insoluble fraction.

Sample	Si (wt%) ^a^	Ca (wt%) ^a^	P (wt%) ^a^	Mg (wt%) ^a^	Sr (wt%) ^a^	F (ppm) ^b^	Ca/P (mol) ^a^
Whole toothpaste	3.5 ± 0.1	1.1 ± 0.1	0.8 ± 0.1	0.02 ± 0.01	0.09 ± 0.01	1458 ± 25	0.96 ± 0.05
Water-insoluble fraction	29.8 ± 1.9	9.2 ± 0.3	4.6 ± 0.2	0.20 ± 0.01	0.72 ± 0.01	1390 ± 25	1.51 ± 0.01

^a^ Quantified by inductively coupled plasma atomic emission spectrometer (ICP-OES); ^b^ quantified by fluoride ion electrode.

**Table 3:** Semiquantitative energy-dispersive X-ray spectroscopy (EDX) compositional analysis of enamel surface layer.

Sample	Si (wt%)	Ca (wt%)	P (wt%)	Mg (wt%)	Sr (wt%)	F (wt%)	Ca/P (mol)
Enamel, control	0.07 ± 0.01	37.9 ± 0.1	16.4 ± 0.1	0.3 ± 0.1	-	-	1.79 ± 0.01
Enamel, treated	1.2 ± 0.1	35.4 ± 0.1	14.5 ± 0.1	0.2 ± 0.1	0.3 ± 0.1	0.22 ± 0.05	1.89 ± 0.01

## Supplementary Materials

The following are available online at https://www.mdpi.com/1996-1944/13/13/2928/s1, Figure S1: EDX spectra of vacuum-dried whole toothpaste, control specimen (demineralized enamel), and treated specimen (enamel treated with toothpaste); Figure S2: EDX elemental mapping analysis of enamel treated with toothpaste; Table S1: Semiquantitative EDX compositional analysis of the vacuum-dried whole toothpaste.
