# Supplementary material for: Characterization of a Toothpaste Containing Bioactive Hydroxyapatites and In Vitro Evaluation of Its Efficacy to Remineralize Enamel and to Occlude Dentinal Tubules
Source: Materials (Basel). 2020 Jun 30;13(13):2928. doi: 10.3390/ma13132928 (PMC7372420; doi:10.3390/ma13132928)
Supplement: Supplementary file 1 [file materials-13-02928-s001.pdf]

materials

Supplementary Materials

Characterization of a Toothpaste Containing Bioactive Hydroxyapatites and in Vitro Evaluation of Its Efficacy to Remineralize Enamel and to Occlude Dentinal Tubules

Lorenzo Degli Esposti [1], Andrei C. Ionescu [2], Eugenio Brambilla [2], Anna Tampieri [1] and Michele Iafisco [1,*]

[1] Institute of Science and Technology for Ceramics (ISTEC), National Research Council (CNR), Via Granarolo 64, 48018 Faenza, Italy; lorenzo.degliesposti@istec.cnr.it (L.D.E.); anna.tampieri@istec.cnr.it (A.T.)

[2] Oral Microbiology and Biomaterials Laboratory, Department of Biomedical, Surgical, and Dental Sciences, University of Milan, Via Pascal, 36, 20133 Milan, Italy; andreiionescu_40@hotmail.com (A.C.I.); eugenio.brambilla@unimi.it (E.B.)

* Correspondence: michele.iafisco@istec.cnr.it; Tel.: +39-0546-699730

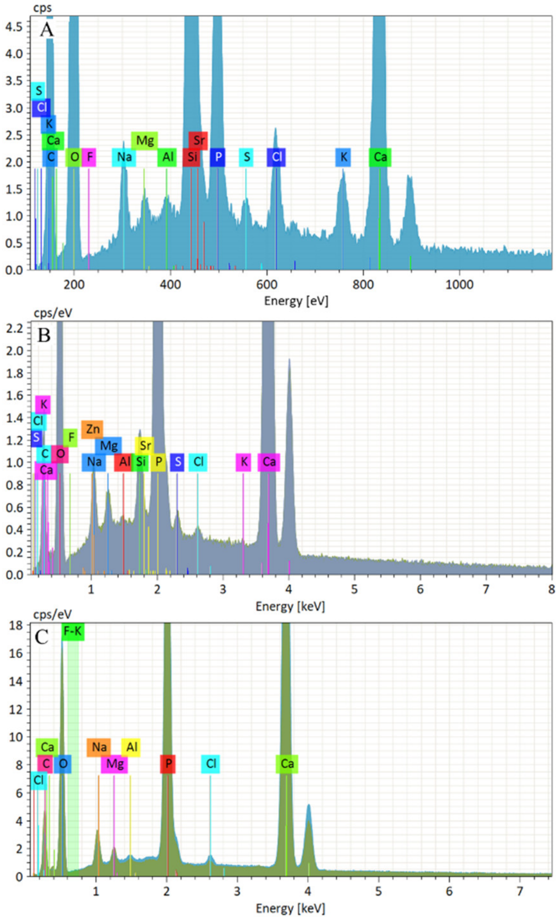

Figure S1. EDX spectra. Example of elemental spectrum of (**A**) vacuum-dried whole toothpaste, (**B**) control specimen (demineralized enamel), and (**C**) treated specimen (enamel treated with toothpaste).

Table S1. Semi-quantitative EDX compositional analysis of the vacuum-dried whole toothpaste.

Sample	Si (wt%)	Ca (wt%)	P (wt%)	Mg (wt%)	Sr (wt%)	F (wt%)	Ca/P (mol)
Whole toothpaste (dry)	12.5 ± 0.1	10.1 ± 0.1	4.7 ± 0.1	0.3 ± 0.1	1.0 ± 0.1	0.12 ± 0.05	1.66 ± 0.02

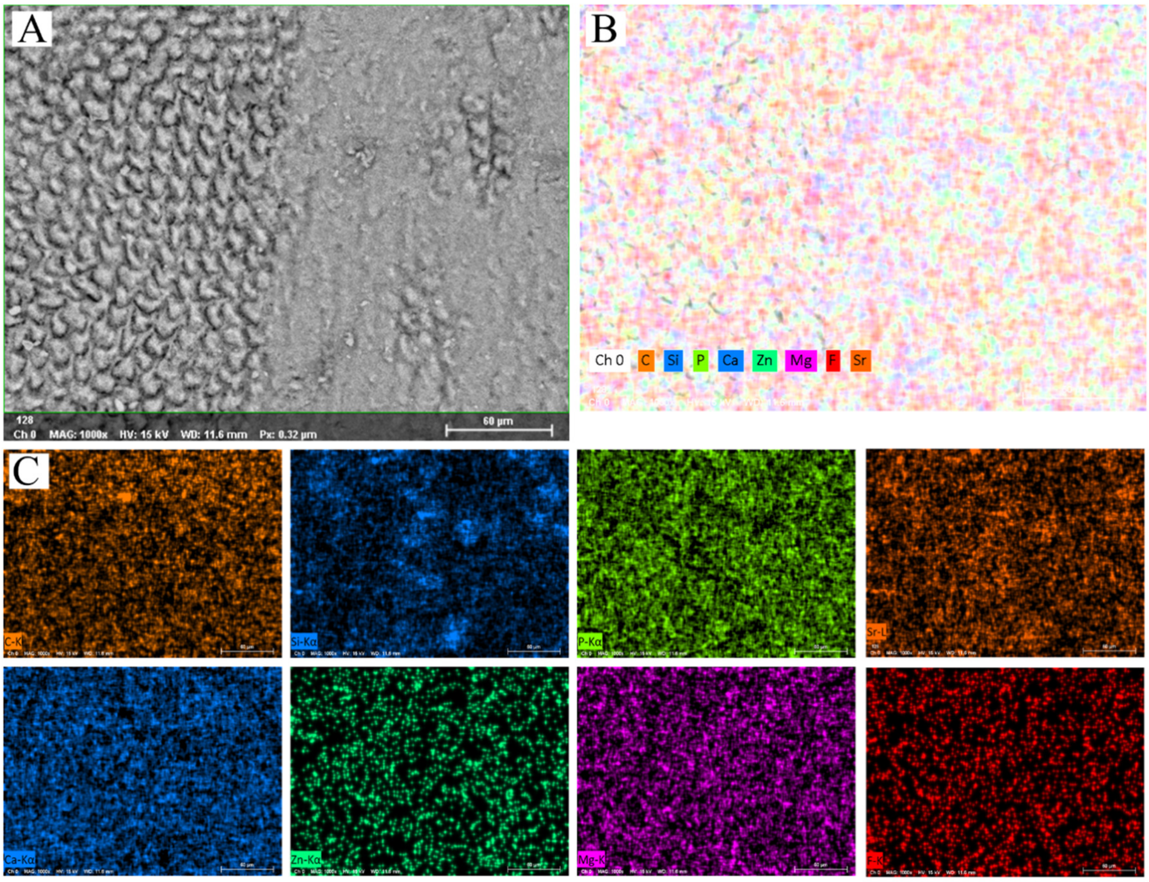

Figure S2. EDX elemental mapping analysis performed on specimens treated with the toothpaste. In (**A**), a field acquired using backscattered electrons is depicted, where the left part of the sample (control) was demineralized and left as is, while the right part was treated with the tested toothpaste after demineralization procedures. Since backscattered electrons are scattered as a function of the atomic number, enamel prisms produce a high signal, while interprismatic enamel that is made by less densely packed crystals and relatively higher carbon content, provide a lower signal. The remineralized layer that was deposited on the surface after toothpaste treatment is clearly detectable. In (**B**), an elemental map of the same region was acquired using the EDS probe. The signal from the single detected elements are shown in (**C**). Clusters of Si particles can be seen having about 30 µm in diameter, that co-localize with Sr signal on the sample part that was treated with the tested toothpaste. No other specific elemental distribution pattern associated with toothpaste deposition could be detected, showing a very similar topographical distribution to pristine enamel, save for the clusters.
